# Combined Inhibition of Akt and mTOR Is Effective Against Non-Hodgkin Lymphomas

**DOI:** 10.3389/fonc.2021.670275

**Published:** 2021-06-18

**Authors:** Ricardo Rivera-Soto, Yi Yu, Dirk P. Dittmer, Blossom Damania

**Affiliations:** ^1^ Curriculum in Genetics and Molecular Biology and Lineberger Comprehensive Cancer Center, The University of North Carolina at Chapel Hill, Chapel Hill, NC, United States; ^2^ ArQule, Inc., A Wholly Owned Subsidiary of Merck & Co., Inc., Kenilworth, NJ, United States; ^3^ Department of Microbiology and Immunology, The University of North Carolina at Chapel Hill, Chapel Hill, NC, United States

**Keywords:** miransertib, MK-4440, non-Hodgkin lymphomas, sirolimus, viral lymphomas

## Abstract

Non-Hodgkin lymphoma (NHL) are a diverse group of hematological malignancies comprised of over 60 subtypes. These subtypes range from indolent to aggressive. The PI3K/Akt/mTOR pathway has been shown to contribute to cell survival and proliferation and is constitutively active in most NHL. MK-7075 (miransertib) and MK-4440 are small molecules that effectively inhibit Akt and have entered clinical development. Using *in vitro* and *in vivo* models of NHL, we explored targeting the kinase Akt with miransertib and MK-4440 alone or in combination with the mTORC1 inhibitor, rapamycin (sirolimus). Both Akt inhibitors inhibited the pathway and NHL proliferation in a subtype-dependent manner. However, these compounds had a minimal effect on the viability of primary B-cells. Importantly, the combination of miransertib and sirolimus synergistically reduced cell proliferation in NHL, including in one indolent subtype, e.g., follicular lymphoma (FL), and two aggressive subtypes, e.g., diffuse large B-cell lymphoma (DLBCL) and primary effusion lymphoma (PEL). To establish *in vivo* efficacy, we used several xenograft models of FL, DLBCL, and PEL. The results obtained *in vivo* were consistent with the *in vitro* studies. The FL xenograft was highly sensitive to the inhibition of Akt alone; however, the tumor burden of PEL xenografts was only significantly reduced when both Akt and mTORC1 were targeted. These data suggest that targeting the PI3K/Akt/mTOR pathway with Akt inhibitors such as miransertib in combination with mTOR inhibitors serves as a broadly applicable therapeutic in NHL.

## Introduction

Lymphomas are a diverse group of neoplastic diseases that arise from lymphoid cells. They are subdivided into Hodgkin and non-Hodgkin lymphomas (NHL). Approximately 90% of lymphoma cases in the United States are classified as NHL, which are further subclassified into >60 subtypes depending on characteristics, such as the cell of origin, stage of cell development, and clinical aggressiveness (1, 2). The American Cancer Society predicts that in the United States alone, >80,000 patients will receive a NHL diagnosis during the year 2021 (2). NHL that arises from B-cells accounts for up to 90% of the cases (1). In this study, we selected multiple different subtypes of NHL to represent the full spectrum of the disease burden, including follicular lymphoma, diffuse large B-cell lymphoma, and primary effusion lymphoma.

Diffuse large B-cell lymphoma (DLBCL) and follicular lymphoma (FL) are the two most common NHL in the United States and responsible for almost half of all NHL diagnoses. FL is characterized by a nodular pattern of growth and develops in the germinal center of lymph nodes (3). Follicular lymphoma is usually indolent, with a median survival of over 15 years. On the other hand, most DLBCL cases tend to present as aggressive diseases requiring immediate intervention. Although most patients respond to current therapeutics, relapses and refractory tumors are not uncommon. Therefore there is a need to develop new therapeutic approaches.

Primary effusion lymphoma (PEL) is an aggressive post-germinal center B-cell lymphoma. It is directly linked to the γ-herpesvirus, Kaposi’s sarcoma-associated herpesvirus (KSHV; HHV8) (4). PEL is characterized by lymphomatous effusions in body cavities such as the peritoneal, pleural, and pericardium that are composed of large plasmablastic cells (5). The disease mostly develops in the context of immunosuppression with a median survival of 6 months (6, 7). PEL are exquisitely dependent on Akt kinase signaling pathways owing to the involvement of viral proteins. This motivated us to target this signaling cascade with novel Akt inhibitors and explore the efficacy of these agents broadly in NHL.

The phosphatidylinositol 3‐kinase (PI3K)/Akt/mammalian target of rapamycin (mTOR) regulates many cellular processes (8–10). Given the significant role that the pathway plays in regulating cell survival, proliferation, and metabolism, it is not surprising to see PI3K/Akt/mTOR signaling deregulated in many cancers (11, 12). The hyperactivation of the pathway can occur through different mechanisms, including mutations or gene amplifications of the kinases PI3K and Akt (also known as protein kinase B), or the loss of the tumor suppressor phosphatase and tensin homolog (PTEN) (13, 14). Viral proteins also activate this pathway substituting for genetic lesions, specifically in PEL (15). Given that multiple NHL subtypes display activated PI3K/Akt/mTOR signaling, members of the pathway have become attractive drug targets. Several compounds advanced through clinical development, e.g., duvelisib, copanlisib, and idelalisib in the treatment of refractory FL, underscoring the clinical significance of targeting this pathway (16–20).

Idelalisib and rapamycin-derivatives (sirolimus) target PI3K and mTOR, but the identification and development of inhibitors for the other kinase in this pathway, Akt, has proven difficult. Recently, two novel pan-Akt inhibitors, miransertib (MK-7075/ARQ 092) and MK-4440 (ARQ 751), have been reported (21). Both compounds are highly specific allosteric inhibitors that target all three Akt isoforms with an inhibitory concentration (IC_50_) as low as 1 nM against purified kinase, and both are orally bioavailable (22). We sought to test these inhibitors against several subtypes of NHL. Our results show that both compounds effectively reduce the viability of several NHL cell lines. Moreover, a combination of miransertib with the allosteric and orally bioavailable, mTORC1 inhibitor sirolimus (rapamycin) resulted in a reduction in tumor growth in four different xenograft models. Overall, this study demonstrates that targeting Akt and mTOR with two individual inhibitors is a feasible approach against these malignancies and warrants clinical development.

## Materials and Methods

### Cell Culture

All non-Hodgkin lymphoma cell lines were cultured on RPMI 1640 (Corning) supplemented with 10% heat-inactivated FBS, 1% penicillin/streptomycin, and 1% L-glutamine. All PEL cells were maintained as described above but further supplemented with 0.075% sodium bicarbonate (Gibco) and 0.05 mM β-mercaptoethanol (Gibco). The human peripheral blood B-cells were purchased from STEMCELL Technologies. Regular mycoplasma testing is performed on the cell lines. Cell lines were authenticated by high-throughput sequencing.

### Compounds

ArQule, Inc. (a wholly owned subsidiary of Merck & Co., Inc.) provided miransertib (ARQ 092) and MK-4440. Miransertib and dactolisib were also purchased from MedChemExpress. For *in vitro* studies, both compounds were dissolved to 30 mM in DMSO and stored at -20°C protected from light. Sirolimus (rapamycin) was purchased from Selleckchem, dissolved to 10 mM in DMSO, and stored at -80°C. For the *in vivo* studies, twice per week, miransertib was dissolved to 10 mg/ml in 0.01 M phosphoric acid (pH 2.2) and protected from light. Sirolimus was dissolved to 10 mg/ml in 100% ethanol and further diluted on the day of administration to equal parts of 20% PEG-400 and 20% Tween-80 in water (23).

### Immunoblotting

Cells were collected, and lysates were prepared from washed pellets using RIPA lysis buffer (1% NP-40, 150 mM NaCl, 50 mM Tris HCl pH 8.0, 5 mM EDTA, 0.5% Sodium deoxycholate, 0.1% SDS). Lysates from tumors were prepared by homogenizing frozen slices with T-PER^®^ Tissue Protein Extraction Reagent (ThermoScientific) according to the manufacturer’s instructions. Lysis buffers were supplemented with Halt protease/phosphatase inhibitor cocktail (ThermoScientific). Samples were clarified by centrifugation at 16,000 x g for 10-15 minutes, and protein concentration was determined by Bradford (Bio-Rad) or Pierce BCA (ThermoScientific) assays. Lysates were loaded with 4x Laemmli sample buffer (Bio-Rad), resolved on acrylamide SDS-PAGE gels, and transferred onto a nitrocellulose membrane (Amersham). For the pAkt (T308) immunoblot, cells were lysed in 1x Laemmli buffer (2% SDS, 10% glycerol, 60 mM Tris-HCl pH 6.8, 0.01% bromophenol blue, 100 mM DTT, 6 M urea, and 5% 2-mercaptoethanol), sonicated for 15s (4.5s on, 0.5s off, 10% amplitude), and boiled at 100°C for 10 min (24). Primary antibodies against pAkt [(S473; 4060) and (T308; 13038S)], Akt (9272), pFOXO1 (S256; 84192), FOXO1 (2880), pS6K (T421/S424; 9204), S6K (9202), pS6 (S235/6; 4858), S6 (2217), PARP (9542), cleaved-caspase-7 (9491), and α-tubulin (9099) were purchased from Cell Signaling Technologies (CST). All primary antibodies were used at a dilution of 1:1000, except for pS6, S6 and tubulin, which were used at a dilution of 1:4000-5000. The HRP-conjugated primary antibody against β-actin (sc-47778 HRP) was purchased from Santa Cruz Biotechnology and used at a 1:5000-10000 dilution. The secondary HRP-conjugated anti-rabbit (7074) antibody was purchased from CST and used at a 1:2000-5000 dilution.

### CellTiter-Glo Cell Viability Assay

One hundred microliters of a suspension of 1.6 x 10^5^ cells/ml were seeded into a 96-well white plate (Greiner Bio-One). Cells were treated with compounds for up to 72 h, and CellTiter-Glo (Promega) was used to determine cell viability according to the manufacturer’s protocol. Luminescence was measured in a CLARIOstar plate reader (BMG Labtech). All data points were collected in triplicates from at least three experiments.

### Trypan Blue Exclusion Assay

One milliliter of 1.6 x 10^5^ cells/ml was plated in 24-well plates and treated with compounds for up to 72 h. The number of live cells was manually counted by excluding dead cells (stained with trypan blue) using a hematocytometer. All data points on NHL cells were collected in duplicates from at least three experiments. The experiments with primary cells were done with 3-6 biological replicates.

### Caspase-3 Activity Assay

A maximum of 2 x 10^6^ cells in 10 ml of total media was treated for 48-72 h, and the activity of caspase-3 was measured by using ApoAlert Caspase-3 Fluorescent Assay Kit (Takara Bio) according to the manufacturer’s instructions. Fluorescence was measured in a CLARIOstar plate reader (BMG Labtech). All data points were collected from at least three experiments.

### Annexin-V Affinity Assay

Two million cells diluted at a concentration of 2 x 10^5^ cells/ml were treated for 48 h. Four hundred thousand cells were washed twice with 200 µl of FACS buffer (0.5% BSA in PBS) and stained with annexin-V antibodies conjugated to FITC and propidium iodide dye according to the manufacturer’s instructions (Biolegend). Samples were processed on a MACSQuant VYB flow cytometer (Miltenyi Biotec). The analysis was conducted using FlowJo software.

### Xenograft Models

All animal studies were performed under protocols approved by the University of North Carolina Institutional Animal Care and Use Committee.

#### PEL Model

Four to five weeks old female NOD-scid-gamma (NSG) mice were intraperitoneally (i.p.) injected with 200 µl of PBS containing 1.0 x 10^5^ BCBL-1-Trex-RTA-Luciferase cells. After three days, a total of 24 mice were randomly sorted into four study arms, vehicle, miransertib, sirolimus, and the combination of miransertib and sirolimus. Treatment began on day three after injection. Two hundred microliters of miransertib or the vehicle for miransertib were administered *via* oral gavage daily (Monday-Friday) to a final dose of 100 mg/kg of body weight. One hundred microliters of sirolimus or the vehicle for sirolimus were administered through i.p. injection twice a week (Monday and Thursday) to a final dose of 0.375 mg/kg of body weight. Once a week and before dosing, imaging was performed using the Xenogen IVIS-Lumina System (Caliper Life Sciences) by administering, through i.p. injection, 10 μl/gbw of luciferin (PerkinElmer). Images were analyzed using Living Image v4.4. All animals were sacrificed when the tumor burden reached institutional limits.

#### BJAB and Karpas-422 Model

Four to five weeks old female NSG mice were subcutaneously (s.c.) injected with 2.0 x 10^5^ (BJAB) or 2.5 x 10^6^ (Karpas-422) cells in 200 μl of a 1:1 mixture of PBS and Matrigel (Corning). For the BJAB experiment, High-Concentration Matrigel was used. Tumor volume was measured thrice per week using calipers. Once tumors were palpable, mice were randomly assigned to four treatment groups and treated following the same schedule as above. The experiments were ended, and animals were sacrificed when the tumor volume reached institutional limits.

#### FL-18 Model

Four to five weeks old female NSG mice were i.p. injected with 200 μl of anti-asialo GM1 antibody (WAKO) that was freshly reconstituted in 6.67 ml of PBS. Twenty-four hours later, 1 x 10^6^ FL-18 cells (200 μl) in a 1:1 mixture of PBS and Growth Factor Reduced Matrigel (Corning) were s.c. injected. Tumor volume was measured as above. Treatments and harvesting were performed as above.

### Immunohistochemistry

Solid tumors were fixed in 10% formalin vials (Fisher Scientific) for up to 3 days, and sections were processed as previously (25). The pS6 primary antibody was purchased from CST (4858) and used at a dilution of 1:400. The images were obtained with a Leica Microscope (DM4000B) and Zeiss camera (Axiocam 105) and acquired using Zeiss Zen Core. Images were analyzed to calculate the H-index using the Multiplex IHC module from HALO (Indica Labs).

### CompuSyn

To assess the effect of combining miransertib and sirolimus, the combination index (CI) was calculated using the Chou-Talalay method (26). The CI was obtained using CompuSyn (http://www.combosyn.com/) according to their instructions. A synergistic effect was determined when the calculated CI at a Fa_50_ (effect level of 50%) was <1.0.

### Statistics

All statistical tests were performed using GraphPad Prism8. The EC_50_ was calculated using the *log(inhibitor) vs. response - Variable slope (four parameters)* function of GraphPad with the data collected after 72 h treatment.

## Results

### Follicular Lymphoma Cell Lines Are Sensitive to Akt Inhibitors

**Figure 1:**
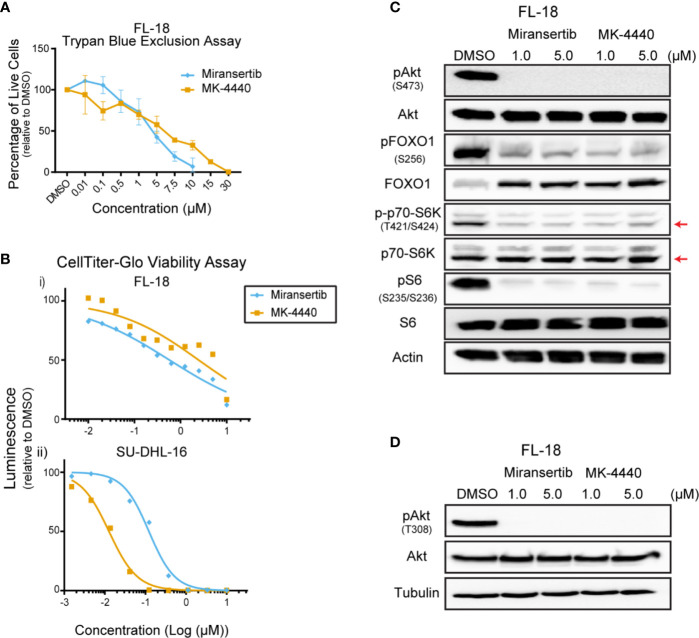
Follicular lymphoma cells are sensitive to Akt inhibition. **(A)** The follicular lymphoma (FL) cell line FL-18 was treated for 72 h with miransertib or MK-4440, and live cells were counted by excluding dead cells stained with trypan blue. **(B)** Two FL cell lines, FL-18 (i) and SU-DHL-16 (ii), were treated as in **(A)**, but viability was measured using CellTiter-Glo. **(C)** FL-18 cells were treated with 1 or 5 µM of miransertib or MK-4440 for up to 72 h. Immunoblots were performed to determine the levels of phosphorylated (p)Akt (S473), pFOXO1 (S256), p-p70-S6K (T421/S424), pS6 (S235/6), the respective total proteins, and the loading control β-actin. The red arrow points to the p70-S6K isoform. **(D)** FL-18 cells were treated with 1 or 5 µM of miransertib or MK-4440 for 24 h. Immunoblots were performed to determine the levels of pAkt (T308), Akt, and the loading control α-tubulin.

To assess the effect of the Akt inhibitors miransertib and MK-4440, we treated a representative FL cell line, FL-18, for 72 h and manually counted live cells using trypan blue. Both compounds reduced the number of live cells in a dose-dependent manner (**Figure 1A**). To determine the half-maximal effective concentration (EC_50_), we performed CellTiter-Glo assays. Treatment with both inhibitors reduced the viability in a dose-dependent manner (**Figure 1B**). **Table 1** shows the calculated EC_50_ obtained from the drug response curves. These results indicate that both compounds were active against FL.

**Table 1:** Summary of EC_50_ for miransertib and MK-4440 calculated in a variety of NHL cell lines.

	Cell Line	EC_50_ (µM)	
		Miransertib	MK-4440
**Follicular Lymphoma**	FL-18	0.573	2.46
	SU-DHL-16	0.122	0.013
**Diffuse Large B-Cell Lymphoma**	BJAB	1.06	1.08
	SU-DHL-4	0.080	0.007
	Karpas-422	0.124	0.012
	WSU-NHL	1.93	0.418
**Primary Effusion Lymphoma**	BC-1	0.837	1.19
	BC-3	4.24	3.53
	BCBL-1	2.28	1.53
	JSC-1	2.33	2.19
	VG-1	0.567	0.258
**Mantle Cell Lymphoma**	Granta-519	2.48	25.77
	Jeko-1	4.46	13.0
	JVM-2	0.817	0.382
	Rec-1	4.84	12.7
**Burkitt’s Lymphoma**	BL-41	1.07	0.711
	Daudi	0.42	1.43
	Ramos	4.23	3.78
	Raji	2.55	18.0

To confirm that miransertib and MK-4440 inhibit Akt and its downstream targets, we performed immunoblotting of protein lysates collected from FL-18-treated cells. These inhibitors prevent Akt from localizing to the plasma membrane blocking its phosphorylation (22). Therefore, we predicted that treatment with the inhibitors would reduce phosphorylation of Akt and its downstream targets, FOXO1, S6K, and S6. Both S6K and S6 are downstream of mTORC1, whereas FOXO1 is a direct Akt target. Both inhibitors effectively reduced the levels of pAkt in FL-18 cells (**Figures 1C, D**). Furthermore, both inhibitors effectively decreased pFOXO1, pS6K, and pS6, demonstrating that the Akt inhibitors repress the mTORC1 and FOXO1 arms of the PI3K/Akt/mTOR signaling pathway. Together, these results indicate that in FL cells, both Akt inhibitors can decrease the activity of the pathway and reduce cell viability.

### Inhibition of Akt and mtorc1 Is Synergistic Against Follicular Lymphoma

**Figure 2:**
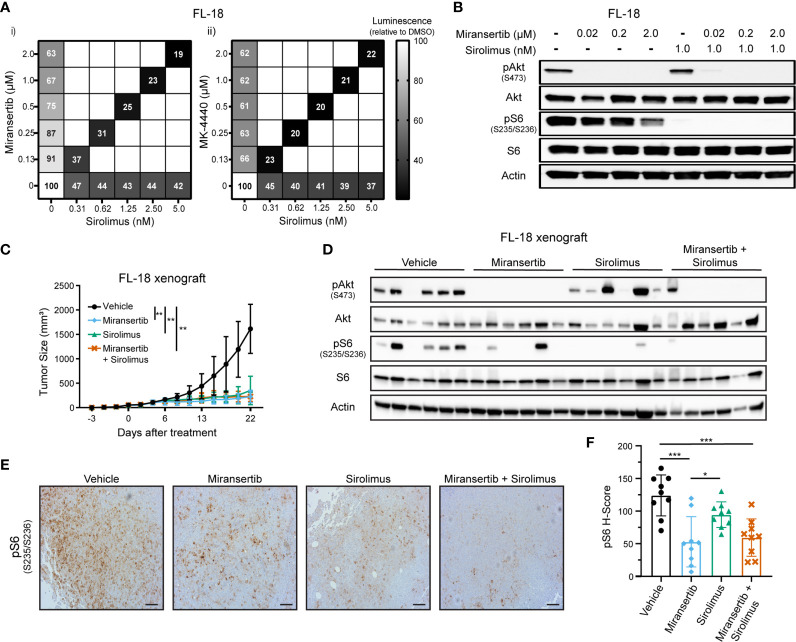
Combined inhibition of Akt and mTORC1 strongly suppresses the growth of follicular lymphoma cells. **(A)** FL-18 cells were treated with different doses of sirolimus alone or in combination with miransertib (i) or MK-4440 (ii). After 72 h, the percentage of viable cells was calculated using CellTiter-Glo, normalized to the DMSO (vehicle; 100%) control, and the heat-map depicts the effect on cell viability. darker color = lower luminescence = lower viability. **(B)** FL-18 cells were treated for 3 h with miransertib, sirolimus, or the combination of both, and immunoblots performed as in **Figure 1C**. **(C)** FL-18 cells were subcutaneously (s.c.) injected into NOD-scid-gamma (NSG) mice and treated with vehicle (n=6), 100 mg/kg miransertib (n=6), 0.375 mg/kg sirolimus (n=6), and 100 mg/kg miransertib + 0.375 mg/kg sirolimus (n=6). The tumor growth was measured thrice per week, and the mean ± STD was illustrated in the line graph. **p < 0.01 determined by two-way ANOVA. **(D)** Immunoblots were performed on proteins extracted from the tumors collected at the end of the experiment shown in panel **(C)**. **(E)** Representative images of immunohistochemistry against pS6 (S235/6) performed to tumor sections. Sections were developed with DAB (3,3′-diaminobenzidine) and counterstained with hematoxylin (Scale bars, 100 μm). **(F)** The histo H-score of pS6 was calculated from 9 frames per treatment group using HALO. *p < 0.05; ***p < 0.001 determined by one-way ANOVA.

Targeting multiple signaling pathways or several members of the same pathway is a common strategy aimed at reducing the chances of developing drug resistance (27). Although we were able to effectively see a reduction in the viability of cells treated with either miransertib or MK-4440, we wanted to know whether the addition of the mTOR inhibitor, sirolimus, would be synergistic. Thus, we measured the viability of FL-18 cells treated with the single compounds miransertib, MK-4440, sirolimus, or the combination of the Akt and the mTORC1 inhibitor (**Figure 2A**). As expected, individual treatment with the three single agents reduced the proliferation of the FL-18 cells. The effect was further enhanced when the Akt and mTORC1 inhibitors were combined. The combination of either miransertib or MK-4440 with sirolimus was strongly synergistic (CI<0.2), according to the Chou-Talalay method (26). For further experiments, we chose to focus on miransertib because it is further along in clinical development.

Another reason to focus on the miransertib/sirolimus combination was the feedback activation of Akt by mTOR. During long, continuous exposure, inhibition of mTORC1 increases the activity of Akt through mTORC2-mediated phosphorylation of serine 473, leading to an increase in activated Akt (14, 28). We predicted that targeting both Akt and mTORC1 would strongly hinder the activation of the overall pathway. To prove this hypothesis, we performed immunoblots for key members of the pathway in lysates collected from singly and dually-treated cells. Treatment with miransertib prevented phosphorylation of Akt and reduced the levels of pS6 (**Figure 2B**). As expected, sirolimus-mediated inhibition of mTORC1 strongly decreased the levels of pS6 while having no effect on pAkt. When cells were treated with both inhibitors, both pAkt and pS6 were reduced. These results demonstrate the superiority of combined inhibition of Akt and mTORC1, which efficiently dampens the entire signaling network.

Next, we tested these inhibitors *in vivo*. NOD-scid gamma (NSG) mice were subcutaneously (s.c.) injected with FL-18 cells. Once the tumors were palpable, mice were treated for three weeks. Treatment with miransertib or sirolimus individually or the combination of both compounds significantly reduced tumor growth (**Figure 2C**). There was no difference between the groups treated with the monotherapy versus the group treated with the combination. At the end of the experiment, tumor sections were lysed, and immunoblotting was performed to verify that the compounds inhibited their target *in vivo*. Treatment with miransertib, but not sirolimus, reduced the levels of pAkt (**Figure 2D**). Furthermore, immunoblots showed that the levels of pS6 were reduced in both miransertib- and sirolimus-treated groups and more completely suppressed in tumors of mice treated with both compounds. Similarly, immunohistochemistry for pS6 performed on tumor sections confirmed a significant reduction in the group treated with the Akt inhibitor alone or in combination with sirolimus (**Figures 2E, F**). These studies demonstrate that in the FL-18 xenograft model, miransertib and sirolimus inhibit their targets and strongly suppress tumor growth.

### Combined Treatment of Miransertib and Sirolimus Represses the Growth of Diffuse Large B-Cell Lymphoma

**Figure 3:**
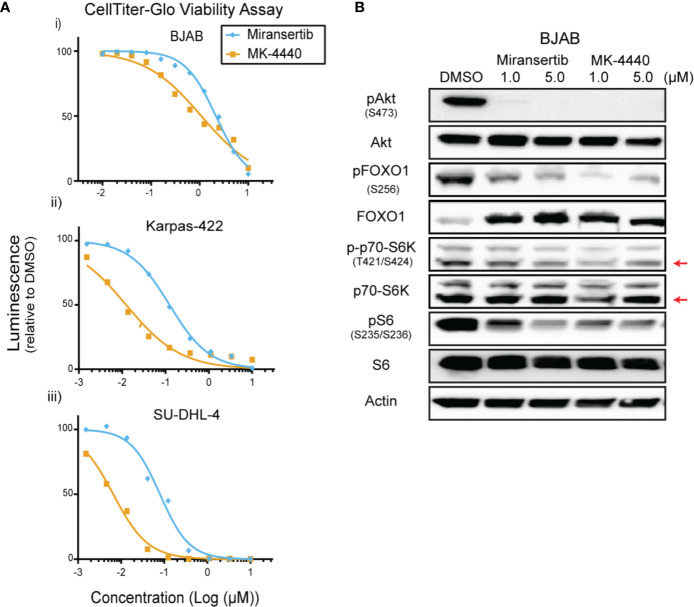
Diffuse large B-cell lymphoma cells are sensitive to Akt inhibition by miransertib and MK-4440. **(A)** The DLBCL cell lines BJAB (i), Karpas-422 (ii), and SU-DHL-4 (iii) were treated for 72 h with miransertib, or MK-4440, and CellTiter-Glo was used to measure viability. **(B)** BJAB cells were treated for up to 72 h with miransertib, sirolimus, or the combination of both, and immunoblots performed as in **Figure 1C**. The red arrow points to the p70-S6K isoform.

To determine whether this approach was effective in other subtypes of NHL, we tested our Akt inhibitors against multiple DLBCL cell lines. We treated four different cell lines, including BJAB and Karpas-422, with a broad range of concentrations for up to 72 h and performed CellTiter-Glo assays. Miransertib and MK-4440 individually reduced DLBCL viability in a cell line-dependent manner. (**Figure 3A** and **Table 1**). Immunoblots with lysates from a representative DLBCL cell line, BJAB, also showed that treatment with either inhibitor sharply reduced the levels of pAkt while only slightly reducing the downstream targets, pFOXO1, pS6K, and pS6 (**Figure 3B**).

**Figure 4:**
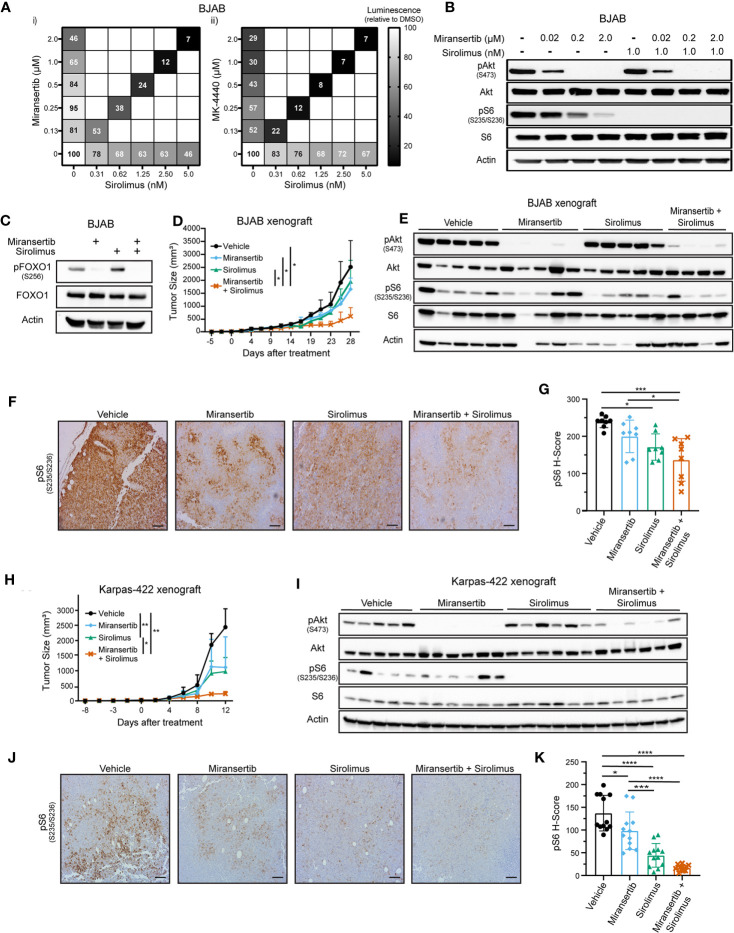
Combined inhibition of Akt and mTORC1 suppresses the growth of diffuse large B-cell lymphoma. **(A)** BJAB cells were treated with different doses of sirolimus alone or in combination with miransertib (i) or MK-4440 (ii). After 72 h, the percentage of viable cells was calculated using CellTiter-Glo, normalized to the DMSO (vehicle; 100%) control, and the heat-map depicts the effect on cell viability. darker color = lower luminescence = lower viability. **(B)** BJAB cells were treated for 3 h with miransertib, sirolimus, or the combination of both, and immunoblots performed as in **Figure 1C**. **(C)** BJAB cells were treated for 48 h with DMSO, miransertib (5 µM), sirolimus (5 nM), or the combination of miransertib and sirolimus. Lysates were prepared, and immunoblots were performed as in **Figure 1C**. **(D)** BJAB cells were s.c. injected into NSG mice, followed by treatment with vehicle (n=6), 100 mg/kg miransertib (n=6), 0.375 mg/kg sirolimus (n=6), and 100 mg/kg miransertib + 0.375 mg/kg sirolimus (n=6). The tumor growth was measured thrice per week, and the mean ± STD was illustrated in the line graph. *p < 0.05 determined by two-way ANOVA. **(E)** Immunoblots were performed from protein extracted from the tumors collected at the end of the experiment shown in **(D)**. **(F)** Representative immunohistochemistry images against pS6 (S235/6) performed on tumor sections as in **Figure 2E**. **(G)** The H-score of pS6 was calculated from 8 frames per treatment group using HALO. *p < 0.05; ***p < 0.001 determined by one-way ANOVA. **(H)** Xenograft with Karpas-422 was performed as in **(D)** *p < 0.05; **p < 0.01 determined by two-way ANOVA. **(I)** Similar to **(E)** but with samples collected from the experiment shown on **(H)**. **(J)** Representative images of immunohistochemistry against pS6 (S235/6) performed on tumor sections as in **Figure 2E**. (Scale bars, 100 μm) **(K)** The H-score of pS6 was calculated from 12 frames per treatment group using HALO. *p < 0.05; ***p < 0.001; ****p < 0.0001 determined by one-way ANOVA.

The differences in reducing the phosphorylation between pAkt and its downstream targets suggest that although Akt is inhibited, the pathway was not entirely repressed. To determine whether combining the Akt inhibitors with the mTORC1 inhibitor, sirolimus, would be effective, BJAB cells were treated for 72 h with single compounds or their combination. Miransertib and MK-4440 each reduced viability to as low as 29% when compared with DMSO (**Figure 4A**). Sirolimus reduced the growth of BJAB cells as well. The combination of either miransertib or MK-4440 with sirolimus was strongly synergistic, with a CI below 0.2. Combining miransertib with sirolimus completely abrogated the phosphorylation of both Akt and S6 (**Figure 4B**). Furthermore, although treatment with sirolimus increased the levels of pFOXO1, this was circumvented when cells were treated with the combination (**Figure 4C**). BJAB serves as an example of an extremely robust DLBCL. These results suggest that for such tumor subtypes, the combined inhibition of Akt and mTORC1 is needed to shut down the PI3K/Akt/mTOR network.

Next, we tested these compounds *in vivo* using two DLBCL xenograft models. NSG mice were s.c. injected with BJAB or Karpas-422 cells. Treatments began once the tumors were palpable and continued until the tumor masses reached institutional limits. The combined Akt and mTORC1 inhibitors treatment inhibited BJAB tumor growth compared with the vehicle or the monotherapy (**Figure 4D**). BJAB tumors were subjected to immunohistochemistry and immunoblotting, which showed that treatment with miransertib alone or in combination with sirolimus reduced the levels of both pAkt and pS6 (**Figures 4E–G**).

Like the BJAB xenograft, treatment with miransertib and sirolimus reduced Karpas-422 tumor growth (**Figure 4H**). Treatment with miransertib alone reduced tumor growth but did not reach statistical significance despite a significant reduction in the levels of pAkt (**Figure 4I**). By contrast, the combination of miransertib and sirolimus strongly and significantly reduced tumor growth and decreased the activation of the pathway as seen by immunoblotting (**Figure 4I**) and immunohistochemistry (**Figures 4J, K**). These results suggest that while DLBCL are sensitive to Akt inhibition by miransertib, more complete therapeutic efficacy is achieved in combination with an mTORC1 inhibitor.

### Miransertib and MK-4440 Repress the Growth of KSHV-Associated Primary Effusion Lymphoma

**Figure 5:**
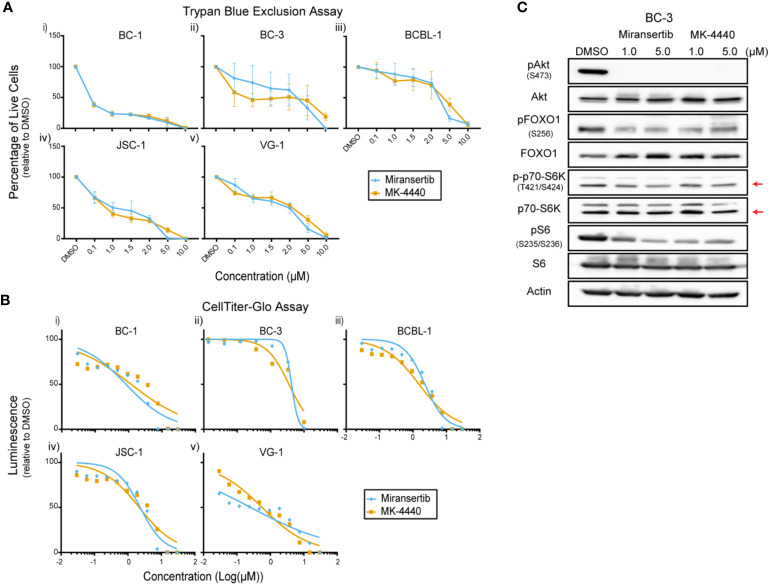
Inhibition of Akt by miransertib or MK-4440 reduces the viability of primary effusion lymphoma cells. **(A)** The PEL cell lines BC-1 (i), BC-3 (ii), BCBL-1 (iii), JSC-1 (iv), VG-1 (v) were treated for 72 h with miransertib, or MK-4440, and live cells were counted by excluding dead cells stained with trypan blue. **(B)** The PEL cell lines BC-1 (i), BC-3 (ii), BCBL-1 (iii), JSC-1 (iv), VG-1 (v) were treated for 72 h with miransertib, or MK-4440, and CellTiter-Glo was used to measure viability. **(C)** A representative PEL cell line, BC-3, was treated for 24 h with miransertib or MK-4440, and immunoblots were performed as in **Figure 1C**. The red arrow points to the p70-S6K isoform.

Primary effusion lymphoma (PEL) is a rare but highly aggressive NHL linked to the γ-herpesvirus Kaposi’s sarcoma-associated herpesvirus (KSHV) (4). The activation of the PI3K/Akt/mTOR signaling pathway is essential for PEL (15, 23, 29, 30). Notably, the KSHV proteins ORF K1, viral G-protein-coupled receptor, and viral interleukin-6 induce the activation of this signaling pathway. Since most PEL cell lines do not display PI3K activating mutations, viral proteins are hypothesized to drive the constitutive activation of the pathway (31). We treated a panel of PEL cells with miransertib or MK-4440 and counted live cells by excluding dead cells stained with trypan blue. Treatment with the inhibitors suppressed growth with none to minimal cells alive at 10 µM (**Figure 5A**). To test a broader range of concentrations and to calculate the EC_50_, we used CellTiter-Glo following a 72-h incubation with the inhibitors. The response to the compounds was complete but varied across different PEL cell lines (**Figure 5B** and **Table 1**). These results demonstrate that Akt inhibitors repress PEL cell growth.

Immunoblots were performed to determine if the compounds were inhibiting their predicted molecular targets. Treatment of BC-3 cells with either compound for 24 h strongly reduced the phosphorylation of Akt but had only minimal effect on the downstream targets pFOXO1, pS6K, and pS6 (**Figure 5C**). This was surprising and underscored the need in certain NHL for inhibiting multiple steps in the same pathway.

### Miransertib Synergizes With Sirolimus to Reduce Tumor Growth in a PEL Xenograft Model

**Figure 6:**
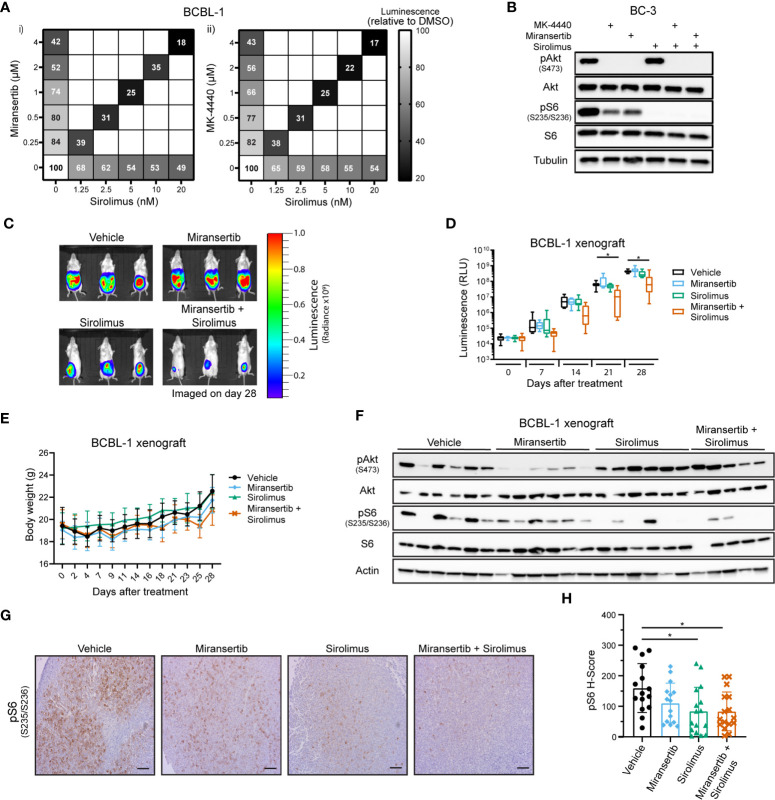
Combined inhibition of Akt and mTORC1 is synergistic against primary effusion lymphoma cells. **(A)** BCBL-1 cells were treated with different doses of sirolimus alone or in combination with miransertib (i) or MK-4440 (ii). After 72 h, the percentage of viable cells was calculated using CellTiter-Glo, normalized to the DMSO (vehicle; 100%) control, and the heat-map depicts the effect on viability. darker color = lower luminescence = lower viability. **(B)** BC-3 cells were treated for 6 h with DMSO, MK-4440 (1.5 µM), miransertib (1.5 µM), sirolimus (5 nM), or the combination of the mTORC1 and Akt inhibitors. Lysates were prepared, and immunoblots were performed as in **Figure 1C**. **(C)** BCBL-1-Luc we intraperitoneally (i.p.) injected into NSG mice and treated with vehicle (n=6), 100 mg/kg miransertib (n=6), 0.375 mg/kg sirolimus (n=6), and 100 mg/kg miransertib + 0.375 mg/kg sirolimus (n=6) over a course of 4 weeks. The expression of luciferase was measured every Monday by i.p. injecting mice with luciferin. Representative images obtained on the 28^th^ day of treatment show the tumor burden. **(D)** The luminescence measured from **(C)** images was presented as a box and whiskers plot with the whiskers marking the minimum and maximum values. *p < 0.05 determined by two-way ANOVA. The p-values for all other comparisons were >0.05. **(E)** The body weight of mice was measured and recorded throughout the experiment. **(F)** Immunoblots were performed from protein extracted from the tumors collected at the end of the xenograft experiment shown on **(C, D)**. **(G)** Representative images of immunohistochemistry against pS6 (S235/6) performed to tumor sections as in **Figure 2E**. (Scale bars, 100 μm) **(H)** The H-score of pS6 was calculated from at least 15 frames per treatment group using HALO. *p < 0.05 determined by one-way ANOVA.

The previous observations suggested that in the PEL subtype of DLBCL, even though Akt was inhibited, the pathway remained active. To overcome this barrier, we tested whether the combination of inhibitors has a synergistic effect on viability. A representative PEL cell line, BCBL-1, was treated with a range of concentrations of either miransertib, MK-4440, sirolimus, or the combination of sirolimus and the Akt inhibitor. Both Akt inhibitors reduced viability compared to DMSO (**Figure 6A**), as did sirolimus, but the reduction by the single agent was moderate (~ 50%). In contrast, when miransertib or MK-4440 was combined with sirolimus, viability reduced by up to 83% after 72 h of treatment. The calculated CI was below 0.2, suggesting that the combination was strongly synergistic. The cell viability results were mirrored by the molecular target analyses. The addition of sirolimus led to a complete reduction in pS6 and in combination with miransertib reduction in pAkt (**Figure 6B**).

To determine if the Akt inhibitor miransertib alone or combined with sirolimus can attenuate tumor growth *in vivo*, we used a PEL xenograft model. To gain more granularity, we followed tumor growth over time using cells expressing a luciferase gene. TRex-RTA BCBL-1 cells were intraperitoneally (i.p.) injected into NSG mice, allowing us to visualize the tumor growth upon injection of luciferin (25, 32). After four weeks of treatment, the combination of miransertib and sirolimus significantly reduced growth compared to the vehicle (**Figures 6C, D**). At the concentrations used, the single agents did not retard growth, mirroring the results from tissue culture experiments. Additionally, we measured the animal weight throughout the experiment to assess the well-being of the animals and determine any *in vivo* toxicity. Generally, animals did not show signs of toxicity and did not suffer weight loss (**Figure 6E**). Tumors collected from mice treated with the combinatorial treatment had lower levels of pS6 (**Figures 6F–H**). Overall, these results suggest that the combined inhibition by miransertib and sirolimus is needed to effectively inhibit aggressive NHL, such as PEL.

### Miransertib and MK-4440 Induce Apoptosis in NHL Cells

**Figure 7:**
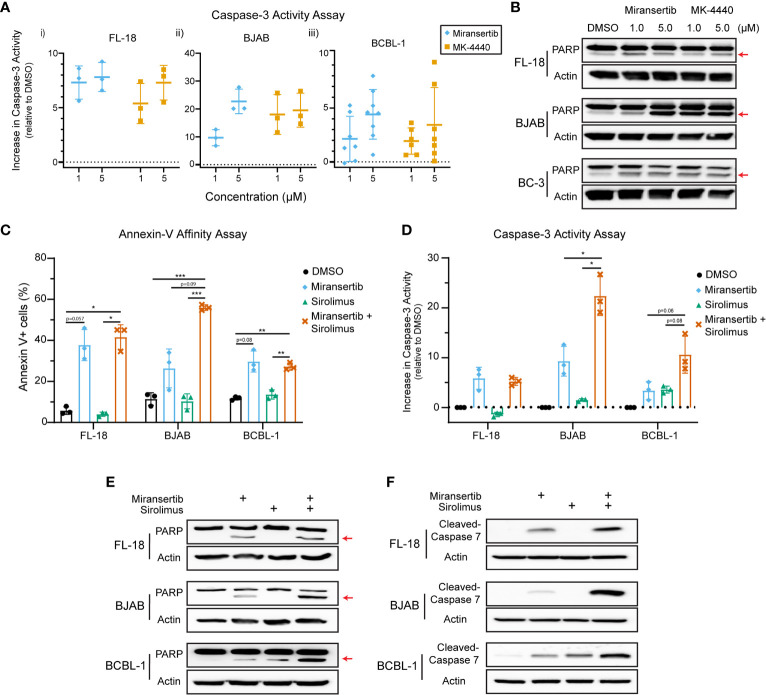
Inhibition of Akt by miransertib or MK-4440 induces apoptosis in non-Hodgkin lymphoma cell lines. **(A)** FL-18 (i), BJAB (ii), and BCBL-1 (iii) cells were treated for up to 72 h with DMSO, miransertib, or MK-4440 at the indicated concentrations. The level of caspase-3 activity following the drug treatment was measured using the ApoAlert Caspase-3 Fluorescent Assay, and the results were graphed as a dot plot representing the increase in activity compared to DMSO (activity = 0). **(B)** FL-18, BJAB, and BC-3 cells were treated for 72 h with DMSO, miransertib, or MK-4440 at the indicated concentrations. Immunoblots were performed to detect the levels of PARP and the loading control β-actin. The red arrow points to cleaved PARP. **(C)** FL-18, BJAB, and BCBL-1 cells were treated for 48 h with vehicle, miransertib (5 µM for BJAB and BCBL-1, and 10 µM for FL-18), sirolimus (10 nM) or the combination of the Akt and mTORC1 inhibitor at the indicated concentrations. The percentage of annexin V+ cells was determined by staining cells with annexin V-FITC and propidium-iodide (PI) followed by flow cytometry analysis. The mean ± STD of 3 experiments is depicted in the bar graph. *p < 0.05; **p < 0.01; ***p < 0.001 determined by one-way ANOVA. **(D)** FL-18, BJAB, and BCBL-1 cells were treated for 48-72 h with vehicle, miransertib (1 µM for BJAB and BCBL-1, and 5 µM for FL-18), sirolimus (10 nM) or the combination of the Akt and mTORC1 inhibitor at the indicated concentrations. The caspase-3 assays were performed as in panel **(A)** The mean ± STD of 3 experiments is depicted in the bar graph. *p < 0.05 determined by Welch’s t-test. **(E)** FL-18, BJAB, and BCBL-1 cells were treated for 48 h with DMSO, miransertib (5 µM for BJAB and BCBL-1, and 10 µM for FL-18), sirolimus (5 nM), or the combination of both inhibitors. Immunoblots were performed to detect the levels of PARP and the loading control β-actin. The red arrow points to cleaved PARP. **(F)** FL-18, BJAB, and BCBL-1 cells were treated as in **(E)**, and immunoblots were performed for cleaved-caspase 7 and the loading control β-actin.

The studies presented above demonstrate that inhibition of Akt with miransertib or MK-4440 reduces the growth of NHL. To determine whether treated cells died or were simply growth-arrested, we measured apoptosis. First, a caspase-3 assay to measure the protease activity was performed. The results show that treatment for 48-72 h with either miransertib or MK-4440 increased the levels of active caspase-3 in FL-18- (i), BJAB- (ii), and BCBL-1- (iii) treated cells (**Figure 7A**). To complement the caspase-3 activity assays, we performed immunoblots to determine the levels of a protein, poly (ADP-ribose) polymerase (PARP), which is cleaved during the induction of apoptosis and thus serves as an apoptotic marker. Similar to the results from the caspase-3 assay, inhibition of Akt led to an increase in the cleavage of PARP in all three lymphoma subtypes (**Figure 7B**). These results suggest that both Akt inhibitors induce apoptosis in NHL cells.

Furthermore, our previous observations suggested that the combination of miransertib and sirolimus is strongly synergistic in reducing the viability of several NHL cell lines. Therefore, we asked whether the combination had a more robust induction of apoptotic signaling. First, we used flow cytometry to quantify the percentage of cells staining for the apoptotic marker, annexin-V. Interestingly, although treatment with miransertib increased the numbers of cells staining for annexin-V, statistical significance was only attained when cells were treated with both Akt and mTORC1, even though sirolimus alone is not an inducer of apoptosis (**Figure 7C**). This observation prompted us to determine the activity of caspase-3 in cells treated with miransertib and sirolimus. We found that in BJAB and BCBL-1 cells, the combination increased caspase-3 activity compared to the single compounds (**Figure 7D**). In the case of FL-18 cells, the combination did not increase the activity beyond the levels seen with miransertib alone. Interestingly, immunoblots demonstrated that in cells treated with the combination of miransertib and sirolimus, there is an increase in the levels of cleaved PARP (**Figure 7E**) and cleaved caspase-7 (**Figure 7F**). Altogether, these results suggest that the reduction in viability is mediated in part *via* apoptosis.

### Miransertib and MK-4440 Are Not Toxic to Primary Human B-Cells

**Figure 8:**
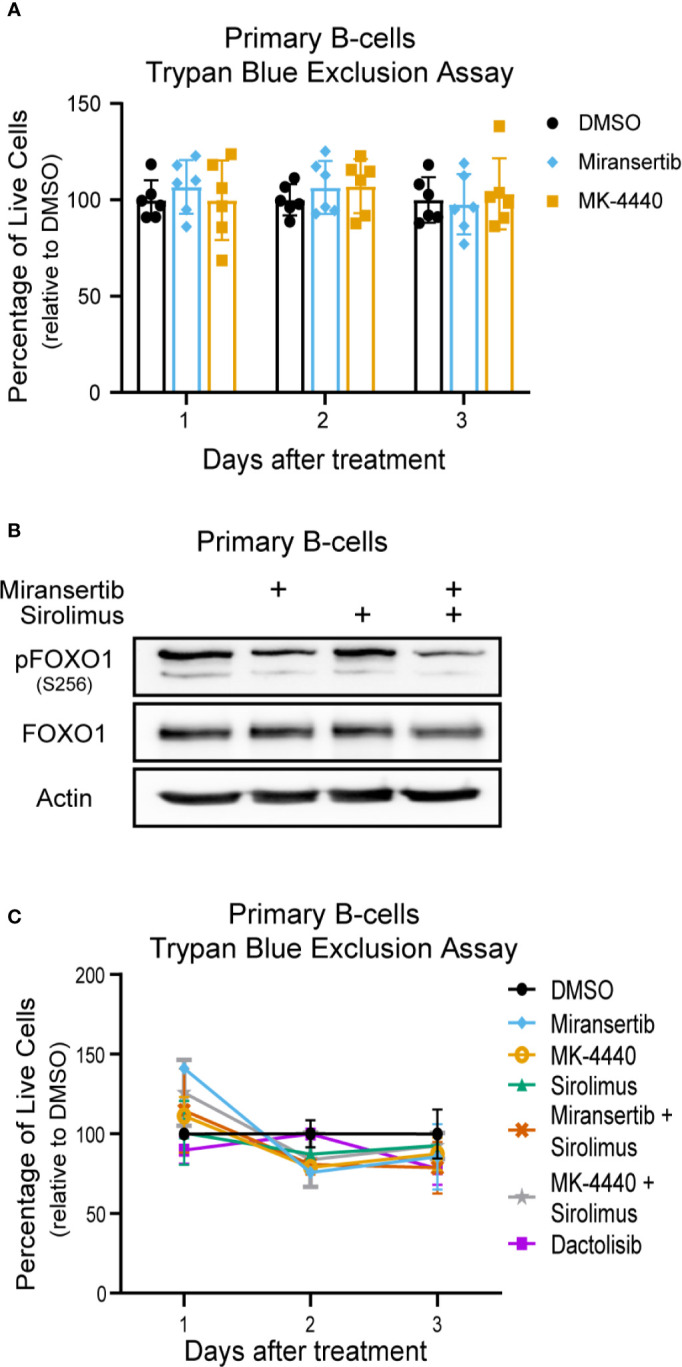
Miransertib and MK-4440 are not toxic to human primary B-cells. **(A)** Human primary B-cells were treated with DMSO, miransertib (2.5 µM), or MK-4440 (2.5 µM), and the number of live cells was manually counted by excluding dead cells stained with trypan blue. Every dot represents one of six biological replicates. **(B)** Primary B-cells were treated for 24 h with DMSO, miransertib (2.5 µM), sirolimus (5 nM), or the combination of both inhibitors. Immunoblots were performed to detect pFOXO1, FOXO1, and the loading control β-actin. **(C)** Human primary B-cells were treated with DMSO, miransertib (0.31 µM), MK-4440 (0.31 µM) sirolimus (0.13 nM), miransertib (0.31 µM) + sirolimus (0.13 nM), MK-4440 (0.31 µM) + sirolimus (0.13 nM), or dactolisib (6.5 nM), and the number of live cells were manually counted by excluding dead cells stained with trypan blue.

Finally, we wanted to determine the effect of the compounds on normal (non-cancerous) cells. To test whether the compounds were toxic to primary cells, we obtained peripheral human B-cells, treated them with DMSO or the compounds, and determined viability by performing trypan blue exclusion assays. The results showed that the compounds had a minimal effect on the viability of primary B-cells (**Figure 8A**). Interestingly, the cells were treated at 2.5 µM, which is higher than the EC_50_ calculated on most cells. Furthermore, to determine whether the inhibitors reduced the activation of the pathway in primary B-cells, cells were treated for 24 h with miransertib, sirolimus, or the combination, and immunoblots were performed for pFOXO1. The results showed that while sirolimus has no effect on pFOXO1, miransertib alone or in combination with sirolimus has only a slight reduction in the phosphorylation of the protein (**Figure 8B**). Finally, we wanted to determine whether the combination of miransertib and sirolimus had a more substantial cytotoxic effect on primary B-cells. To do that, we treated primary B-cells with the Akt inhibitors and sirolimus alone or in combination at a concentration where the combination was enough to reduce viability to about 50% in NHL cells (**Figures 2A**, **4A**, **6A**). The results demonstrated that in contrast to NHL cells, the combination did not strongly reduce the viability of normal cells (**Figure 8C**). Interestingly, in terms of viability, the combination of miransertib and sirolimus had a similar effect to those observed with the dual PI3K/mTOR inhibitor, dactolisib. Altogether, these results suggest that the compounds might have a more potent effect on NHL cells, which might be due to their addiction to the PI3K/Akt/mTOR pathway.

## Discussion

**Figure 9:**
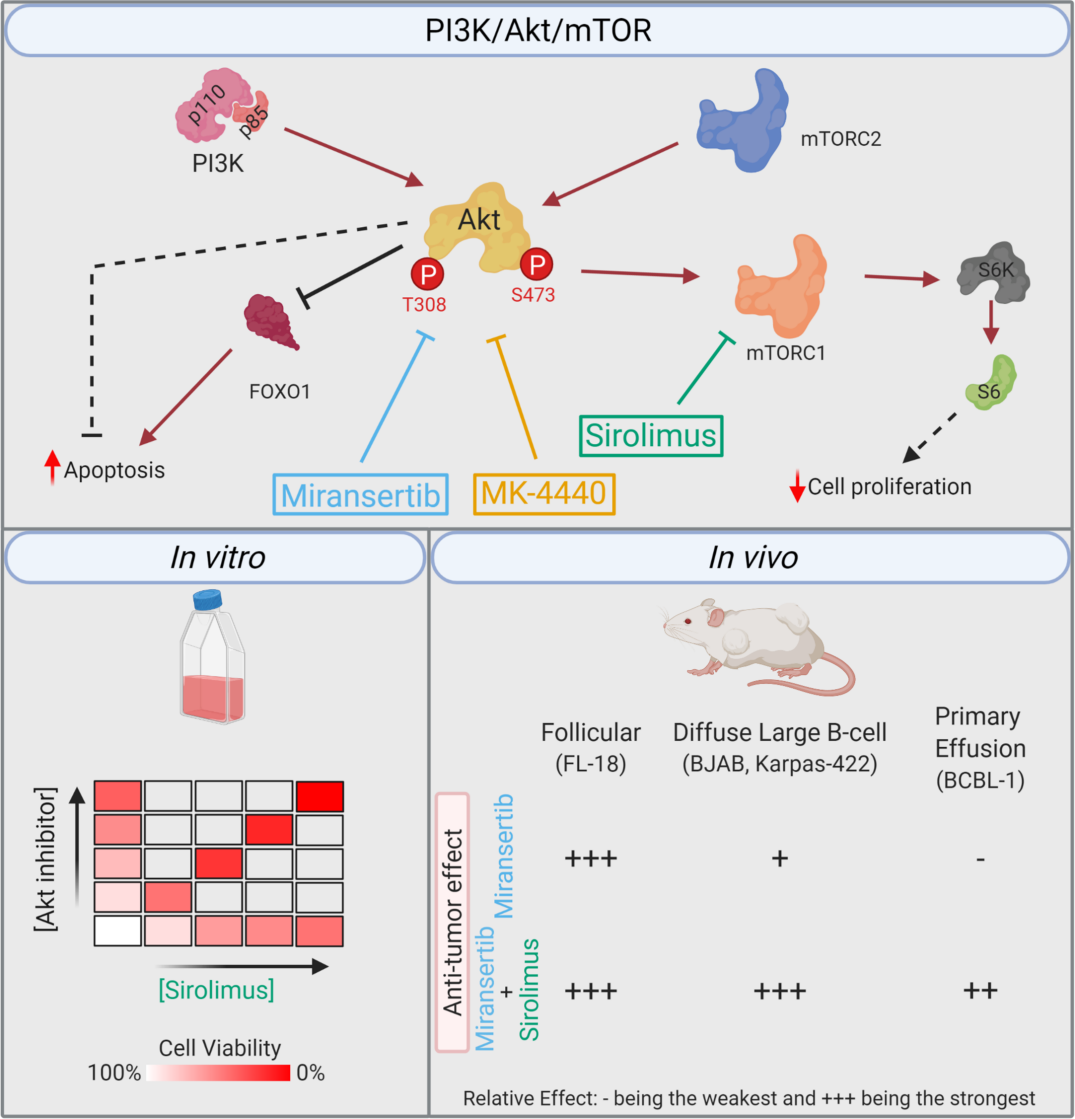
Summary. Simplified model of the PI3K/Akt/mTOR pathway, including the compounds and their respective target. The *in vitro* studies demonstrated that the combination of miransertib or MK-4440 and sirolimus was synergistic against a variety of NHL cell lines. In the *in vivo* studies, the follicular lymphoma model was highly sensitive to miransertib treatment alone. The diffuse large B-cell lymphoma models were slightly sensitive to miransertib, but the addition of sirolimus strongly repressed tumor growth. For the highly aggressive primary effusion lymphoma model, only mice treated with the combination of Akt and mTOR inhibitors suppressed tumor growth (created with BioRender.com).

The numerous therapeutical approaches used to treat NHL demonstrate the heterogeneity of these malignancies and the lack of a broadly efficacious therapy. The continued discovery of molecular targets and novel treatments is vital to improving overall survival for this disease cluster. One signaling pathway that has attracted attention in NHL is the PI3K/Akt/mTOR network (**Figure 9**) (12). Supraphysiological activation of this pathway can result from activating mutations in the PI3K isoforms and Akt, the loss of the negative regulator PTEN and the introduction of viral oncogenes. In addition to being vital to the survival of cancer cells, this signaling pathway’s hyperactivation is believed to be an important player in drug resistance (33). For instance, the resistance of activated B-cell DLBCL cell lines to the Bruton’s tyrosine kinase inhibitor, ibrutinib, is mediated through overactivation of the PI3K/Akt/mTOR (34, 35). Conversely, PI3K inhibitors synergized with chemotherapy agents against ibrutinib-resistant DLBCL cells. These examples highlight the vital role that this signaling pathway might play as a therapeutic target against NHL.

In recent years, three PI3K inhibitors (duvelisib, copanlisib, and idelalisib) have received approval from the United States’ Food and Drug Administration (FDA) to treat refractory FL and are currently in clinical trials for other NHL subtypes (18–20). An additional PI3Kα inhibitor, alpelisib, in combination with fulvestrant, was approved in 2019 to treat certain breast cancers (36). mTOR belongs to the family of phosphatidylinositol 3-kinase-related protein kinases due to its similarity with PI3K. The pharmacological inhibition of mTOR through rapamycin and its derivatives (rapalogs) has been approved for various treatments, including cancers, and studied in the context of lymphomas. Feng et al., discussed in detail the development of these compounds against hematological malignancies (37). To mention a few, several phase I-II trials have been conducted to test the efficacy of these compounds (e.g., everolimus and temsirolimus) and have shown some efficacy as monotherapy or in combination with other standard chemotherapy regimens such as R-CHOP or lenalidomide (38–41). Furthermore, a recent study demonstrated the efficacy of the combination of the histone deacetylase inhibitor, vorinostat, in combination with sirolimus or everolimus in heavily treated relapsed/refractory lymphoma patients (42). Although mTOR inhibition has shown some clinical value, the development of these compounds for such indications has slowed down as demonstrated by the lack of active phase III trials (37).

Akt inhibitors have lagged behind in discovery and clinical development. An extensive literature review on the development of Akt inhibitors was recently published by Song et al. (43). Some of the most promising candidates include the ATP-competitive Akt inhibitors capivasertib (AZD5363) and ipatasertib (GDC-0068), and the allosteric inhibitor MK-2206. All three compounds have progressed through clinical trials with mixed results (44–46). Several phase III trials are ongoing using capivasertib combined with compounds such as paclitaxel, abiraterone, palbociclib, or fulvestrant for the treatment of a variety of conditions, including breast cancers (NCT03997123; NCT04862663; NCT04305496) and prostate cancers (NCT04493853). Notably, some clinical data has demonstrated that patients with aberrant PI3K/Akt/mTOR tend to show the best response highlighting the pathway as a possible indicator for therapy selection (44). On the other hand, although Roche’s ipatasertib in combination with paclitaxel had promising phase I-II data as first-line treatment for metastatic triple-negative breast cancer, in phase III trials, the combination failed to reach the primary endpoint (47, 48). Similarly, MK-2206 has demonstrated a manageable safety profile but has, for the most part, failed to show clinical efficacy, including in refractory lymphoma patients (49). The encouraging results seen with capivasertib demonstrate the likely clinical benefit in targeting Akt. Therefore, significant research effort should be centered on characterizing novel compounds.

Miransertib is one of the most recent Akt inhibitors to enter clinical trials. Given the importance of Akt, which is commonly known as the “cell survival kinase,” we explored miransertib for its therapeutic benefits against NHL. Some pre-clinical and early clinical studies have demonstrated the efficacy of the orally bioavailable pan-Akt inhibitor miransertib against certain cancers (22, 50, 51). Preliminary results of a phase I trial in heavily pretreated NHL, and chronic lymphocytic leukemia patients showed that 3/11 patients had a partial response to miransertib (51). Moreover, miransertib is currently undergoing a phase II trial (NCT04316546) to treat a rare but progressive overgrowth syndrome (Proteus syndrome) that arises from the E17K mutation in Akt, with case reports showing single-agent activity (52–54). Notably, no toxicities were reported in these case studies, which included pediatric cases. Thus, we wanted to test whether the Akt inhibitors miransertib and MK-4440 are effective against NHL spanning indolent to aggressive forms. We found that both Akt inhibitors reduced the activation of the PI3K/Akt/mTOR signaling pathway, decreased cell proliferation, and had efficacy in xenograft models. The effect was subtype-dependent. Some cell lines were more drug-resistant than others. Cell lines representing mantle cell lymphoma and Burkitt’s lymphoma (BL) were amongst the most resistant (**Table 1**). Our results obtained with BL are consistent with a recent report showing that BL cells grow despite high PTEN levels and are not sensitive to Akt inhibition (24). Most other subtypes, specifically FL, DLBCL, and PEL, were sensitive to Akt inhibition.

Interestingly, although treatment with miransertib or MK-4440 alone strongly inhibited Akt, the pathway remained active as downstream targets were slightly phosphorylated especially in DLBCL and PEL cells. This leftover activation might be responsible for the higher resistance detected in these cells as compared to FL. Previous studies in DLBCL demonstrated that Akt-independent activation of S6K through upregulation of PIM2 or the B-cell receptor is a major driver of resistance to Akt inhibition (55). Furthermore, KSHV viral proteins are believed to be significant drivers of tumorigenesis in PEL cells, and several of these proteins have been found to activate the PI3K/Akt/mTOR signaling pathway (15). One such protein is the product of ORF36, viral protein kinase, which resembles S6K and phosphorylates S6 (56). Similarly, upregulation of interleukin-6 and JAK/STAT signaling was reported as a resistance mechanism against PI3K inhibitors in lymphoma (57). Notably, KSHV encodes for a viral homolog to interleukin-6, which constitutively activates JAK/STAT signaling (58). Therefore, there are multiple avenues for which extrinsic factors such as viral proteins and intrinsic factors such as mutations or overactivation of parallel pathways might drive resistance to Akt inhibition.

The dual PI3K and mTOR inhibitor compound dactolisib (NVP-BEZ235) is broadly effective against NHL, inducing cell death and strongly repressing the PI3K/Akt/mTOR pathway (29, 59); however, the clinical development of dactolisib was terminated due to toxicity (60–62). The EC_50_ of dactolisib against NHL was <10 nM, which is significantly lower than what we observed with miransertib and MK-4440. Furthermore, dactolisib strongly repressed tumor growth at a concentration of ~40 mg/kg, whereas for miransertib, we used 100 mg/kg (29, 59). The experience with dactolisib provides proof of principle that combined inhibition of multiple targets in this pathway is broadly efficacious. Our results support this paradigm. Neither miransertib nor MK-4440 alone completely inactivated the pathway as determined by phosphorylation of S6, but the combined inhibition of Akt and mTOR synergistically reduced cell growth. The advantage of this approach compared to using one compound that targeted two kinases, as in the case of dual inhibitors, is that the dose of each inhibitor, miransertib, or sirolimus, can be adjusted individually to provide maximum benefit and minimum cytotoxicity.

The combined inhibition of mTORC1 and Akt prevented the mTORC2-mediated phosphorylation of Akt at residue 473. This phosphorylation is usually increased upon inhibition of mTORC1 by sirolimus and is predicted to contribute to drug resistance (14, 28). The newer generation of mTORC1 and mTORC2 inhibitors might provide an even more robust effect.

Follicular lymphoma stood out in our survey because this tumor type seemed exquisitely sensitive to even single agents. Here the administration of miransertib or sirolimus significantly reduced the tumor growth in the FL-18 model. The combination of both inhibitors did not prevent tumor growth beyond the effect seen in mice treated with monotherapy, even though the tumors from the dually-treated group expressed the lowest amount of pS6. Unfortunately, *in vitro* models for studying FL are limited as patient-derived cell lines resemble transformed FL (63). The lack of resources in this and other FL studies provides a challenge for collecting pre-clinical data with unadulterated FL.

In the case of DLBCL, both BJAB and Karpas-422 xenografts responded the best when treated with the combination of miransertib and sirolimus. Both immunohistochemistry and immunoblots demonstrated a reduction in the phosphorylation of S6, suggesting that the compounds reached the tumors *in vivo*. Similarly, a substantial reduction in tumor growth in the PEL xenograft was only attained in the arm treated with the combination. Interestingly, although the combination of miransertib and sirolimus reduced pS6 in PEL tumors, it had minimal effect on pAkt. Since the BCBL-1 xenografts have both solid and liquid components, it is possible that our compounds did not completely reach the solid tumors even though it strongly repressed the dissemination of ascites. These observations illustrate the difficulties of treating lymphomas composed of effusions such as PEL.

Several Akt targets are directly involved in the inhibition of apoptosis (64). These targets include the FOXO family of proteins (65). Our results demonstrated that Akt inhibition increased the levels of FOXO1 protein. Thus, we reasoned that miransertib and MK-4440 should trigger the induction of apoptosis, which might be responsible for decreasing cell viability. To analyze the apoptotic state in treated cells, we measured the activity of caspase-3 and the presence of cleaved PARP and caspase-7. These studies demonstrated that inhibition of Akt induced the activation of apoptotic signaling and it was increased when combined with sirolimus. Altogether, inhibition of Akt in several NHL cell lines induces apoptosis and might be responsible for the reduction in cell growth.

In conclusion, this report demonstrates the feasibility and need for targeting Akt and mTORC1 as a treatment for NHL. To our knowledge, this report is the first one that uses a variety of *in vitro* and *in vivo* models to demonstrate the approach against hematological malignancies. Although this study examines miransertib and sirolimus as an all-oral regimen that might achieve this goal, we believe that this strategy should continue to be explored with newer and more specific inhibitors.

## Data Availability Statement

The original contributions presented in the study are included in the article/supplementary material. Further inquiries can be directed to the corresponding author.

## Ethics Statement

The animal study was reviewed and approved by UNC IACUC.

## Author Contributions

RR-S, DD, and BD designed the research, analyzed results, and wrote the manuscript. RR-S performed experiments. YY provided compounds and technical advice. All authors contributed to the article and approved the submitted version.

## Funding

This work and BD was supported by National Institutes of Health (NIH) grants CA096500, CA163217, and CA254564. DD was supported by NIH grants CA239583 and CA228172. RR-S was supported by NIH grants GM007092 and CA019014 and holds a Graduate Diversity Enrichment Program Award from the Burroughs Wellcome Fund.

## Conflict of Interest

YY worked for ArQule, Inc, now a wholly owned subsidiary of Merck & Co., Inc. BD served as an advisor to ArQule, Inc.

The remaining authors declare that the research was conducted in the absence of any commercial or financial relationships that could be construed as a potential conflict of interest.
